# Engineered Mesenchymal Stem Cells Expressing Stromal Cell-derived Factor-1 Improve Erectile Dysfunction in Streptozotocin-Induced Diabetic Rats

**DOI:** 10.3390/ijms19123730

**Published:** 2018-11-23

**Authors:** Seung Hwan Jeon, Guan Qun Zhu, Woong Jin Bae, Sae Woong Choi, Hyun Cheol Jeong, Hyuk Jin Cho, U-Syn Ha, Sung-Hoo Hong, Ji Youl Lee, Eun Bi Kwon, Hyo-Jin Kim, Soon Min Lee, Hey-Yon Kim, Sae Woong Kim

**Affiliations:** 1 Department of Urology, College of Medicine, The Catholic University of Korea, Seoul 06591, Korea; shwan52@naver.com (S.H.J.); xiaoguanqun1@gmail.com (G.Q.Z.); bwoong@catholic.ac.kr (W.J.B.); lifeisa9ame@catholic.ac.kr (S.W.C.); koulich@naver.com (H.C.J.); hyukjincho0403@gmail.com (H.J.C.); ushamd@catholic.ac.kr (U.-S.H.); toomey@catholic.ac.kr (S.-H.H.); uroljy@catholic.ac.kr (J.Y.L.); eunbi9859@naver.com (E.B.K.); 2 Catholic Integrative Medicine Research Institute, The Catholic University of Korea, Seoul 06591, Korea; 3 Department of Biomedicine & Health Sciences, The Catholic University of Korea, Seoul 06591, Korea; 4 Department of Stem cell therapy, SL BIGEN, Seongnam 13488, Korea; hjkim@slbigen.com (H.-J.K.); smlee@slbigen.com (S.M.L.); hykim@slbigen.com (H.-Y.K.)

**Keywords:** stromal cell-derived factor-1 expressing engineered mesenchymal stem cells, erectile dysfunction, intracavernosal injection, streptozotocin-induced diabetic rats

## Abstract

Effective therapies for erectile dysfunction (ED) associated with diabetes mellitus (DM) are needed. In this study, the effects of stromal cell-derived factor-1 (SDF-1)-expressing engineered mesenchymal stem cells (SDF-1 eMSCs) and the relevant mechanisms in the corpus cavernosum of a streptozotocin (STZ)-induced DM ED rat model were evaluated. In a randomized controlled trial, Sprague–Dawley (SD) rats (*n* = 48) were divided into four groups (*n* = 12/group): Normal (control), DM ED (diabetes induced by STZ), DM ED + BM-MSC (treated with bone marrow [BM]-derived MSCs), and DM ED + SDF-1 eMSC (treated with SDF-1-expressing BM-MSCs). After four weeks, intracavernosal pressure (ICP), an indicator of erectile function, was 0.75 ± 0.07 in the normal group, 0.27 ± 0.08 in the DM ED group, 0.42 ± 0.11 in the DM ED + BM-MSC group, and 0.58 ± 0.11 in the DM ED + SDF-1 eMSC group. BM-MSCs, especially SDF-1 eMSCs, improved ED (*p* < 0.05). SDF-1 eMSC treatment improved the smooth muscle content in the corpus cavernosum (*p* < 0.05). As SDF-1 expression increased, ED recovery improved. In the SDF-1 eMSC group, levels of neuronal nitric oxide synthase (nNOS) and phosphorylated endothelial NOS (p-eNOS) were higher than those in other groups (*p* < 0.05). In addition, high stromal cell-derived factor-1 (SDF-1) expression was associated with increased vascular endothelial growth factor (VEGF) and basic fibroblast growth factor (bFGF) in DM ED rats (*p* < 0.05). Higher levels of phosphorylated protein kinase B (p-AKT)/protein kinase B (AKT) (*p* < 0.05) and B-cell lymphoma-2 (Bcl-2) and lower levels of the apoptosis factors Bcl2-associated x (Bax) and caspase-3 were observed in the MSC-treated group than in the DM ED group (*p* < 0.05). SDF-1 eMSCs showed beneficial effects on recovery from erectile function.

## 1. Introduction

Increased living standards have been accompanied by an increased demand for erectile dysfunction (ED) therapy, especially in elderly men who suffer from diabetes mellitus (DM) [1,2]. The main current treatment strategy for ED is phosphodiesterase 5 inhibitor (PDE5I), which affects angiectasis of the corpus cavernosum [3]. However, for ED caused by DM, PDE5I is not sufficiently effective [4,5]. Alternative remedies, such as vacuum constriction devices, have limitations [6]. Accordingly, a new and effective therapeutic method is urgently needed for DM-associated ED.

Mesenchymal stem cells (MSCs) have long-term self-renewal abilities as well as the capacity to differentiate into a variety of cell types in certain conditions [7]. In different physiological and pathological conditions, MSCs could maintain homeostasis by multi-directional differentiation. They secrete large amounts of cytokines, which carry chemical signals between cells, and are widely used in studies of regenerative medicine [8,9,10]. Novel MSC-based therapeutic approaches have shown satisfactory treatment effects in clinical applications. MSC therapy is used for the treatment of nerve injury and trauma, inflammatory disease, and transplantation [11,12,13]. MSCs can promote cellular growth and prevent apoptosis [14,15]. MSC therapy for ED is still in the clinic trial phase [16,17]. Most studies have shown [18,19,20] that MSC therapy has positive effects on ED. However, the precise effects of MSCs on ED and the mechanisms underlying these effects are unclear [21].

Recent studies [22] have shown that in cavernous-nerve injury-induced ED rats with increased stromal cell-derived factor-1 (SDF-1) expression, ED could be improved. Additionally, Yamaguchi et al. [23] suggested that SDF-1 could drive endothelial progenitor cell recruitment and improve angiogenesis. Accordingly, we believe that an effective method to increase SDF-1 expression in the corpus cavernosum is a potential approach for ED treatment. In the corpus cavernosum of patients with DM-associated ED, there are a number of vascular injuries, including vascular endothelial cell and vascular smooth muscle damage [24,25,26]. Vascular repair would be improved by sufficient levels of vascular endothelial growth factor (VEGF) in the corpus cavernosum. Additionally, some studies [27] have suggested that VEGF released by MSCs could recover injured vascular tissues. However, other factors explaining the high VEGF expression and the mechanisms by which MSCs in injured tissues promote VEGF expression are unclear.

In this study, we built an ED streptozotocin (STZ)-induced DM rat model. We evaluated the effects and underlying mechanisms of MSC therapy on this DM ED model after SDF-1-expressing bone marrow MSC (SDF-1 eMSC) transplantation. We hypothesized that transplanted SDF-1 eMSCs could increase angiogenesis and thereby improve ED. The main aim of this study was to explore the effects of high SDF-1 expression via BM-MSC therapy and to investigate the relevant mechanisms in the corpus cavernosum of the STZ-induced DM ED rat model.

## 2. Results

### 2.1. Measurements of Body Weight and Blood Glucose Levels

After STZ injection, body weights were significantly lower in DM rats than in Normal rats. After four weeks, the DM ED + SDF-1 eMSC group had significantly greater (*p* < 0.05) weight gain than the DM ED group and significantly lower blood glucose levels (*p* < 0.05) than the DM group (Table 1).

### 2.2. Stromal Cell-derived Factor-1-Expressing Engineered Mesenchymal Stem Cells Significantly Improve Diabetes Mellitus Erectile Dysfunction

**Figure 1:**
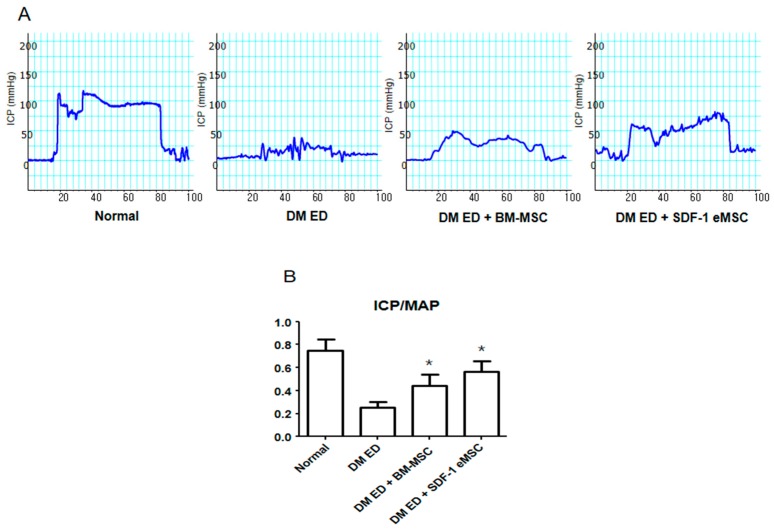
Comparison of erectile function among groups. (**A**) Representative images of intracavernous pressure (ICP) in response to electrical stimulation of the cavernosal nerve. (**B**) Ratio of ICP to mean MAP (mean arterial pressure) in each group. Each bar shows the mean value (standard deviation). * *p* < 0.05 compared with the DM ED (diabetes mellitus erectile dysfunction) group.

Representative images of intracavernosal pressure (ICP) results are shown in Figure 1. The ICP of the DM ED + BM-MSC group was higher than that of the DM ED group. In a quantitative analysis (Figure 1B), the ICP of the normal group was 0.75 ± 0.07, the ICP of the DM ED group was 0.27 ± 0.08, the ICP of the DM ED + BM-MSC group was 0.42 ± 0.11, and the ICP of the DM ED + SDF-1 eMSC group was 0.58 ± 0.11. These results showed that treatment with BM-MSCs, especially SDF-1 eMSCs, could improve ED. The ICP/MAP ratio was significantly higher in the DM ED + BM-MSC and DM ED + SDF-1 eMSC groups than in the DM ED group (*p* < 0.05).

### 2.3. Stromal Cell-derived Factor-1-Expressing Engineered MSCs Improve the Smooth Muscle Content and Angiogenesis in the Corpus Cavernosum

**Figure 2:**
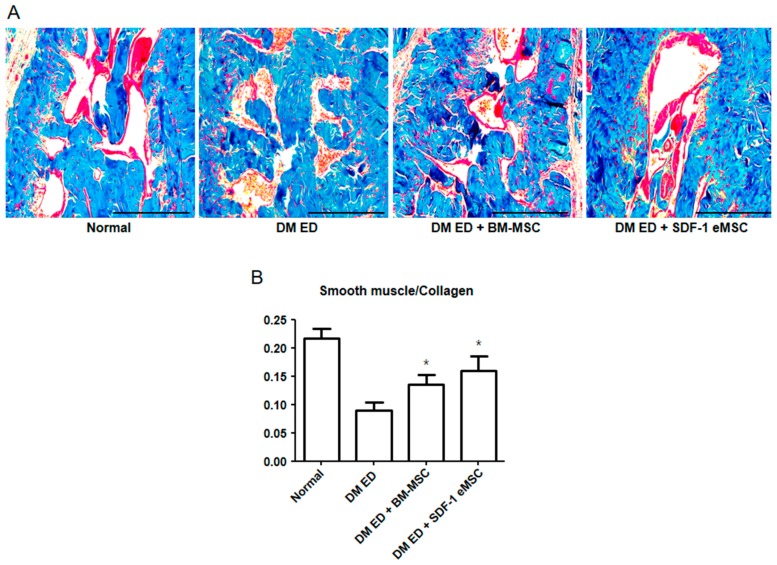
Representative images of Masson Trichrome staining in the corpus cavernosum. (**A**) Red indicates smooth muscle, and blue represents collagen. Scale bar: 100 μm. Original magnification: ×100; (**B**) Percentage area of smooth muscle for each group. Each bar shows the mean value (standard deviation). * *p* < 0.05 compared with the DM ED group.

**Figure 3:**
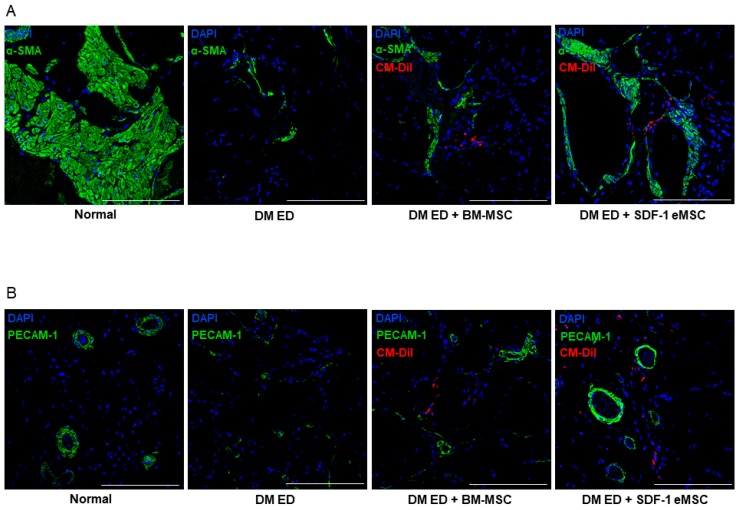
Representative images of immunofluorescence staining in the corpus cavernosum after treatment with mesenchymal stem cells (MSCs). (**A**) Representative images of α-SMA staining for each group. Red indicates MSCs, green represents α-SMA, and blue represents cell nuclei. Scale bar: 100 μm. Original magnification: ×200; (**B**) Representative images of PECAM-1 staining for each group. Red indicates MSCs, green is PECAM-1, and blue represents cell nuclei. Original magnification: ×200.

The smooth muscle and collagen contents in the corpus cavernosum were observed by Masson’s trichrome staining. As shown in Figure 2A, the smooth muscle contents were higher in the DM ED + BM-MSC group than in the DM ED group. These results indicated that as the expression of SDF-1 increased, recovery in the ED rats improved. As shown in Figure 3, after the MSC injection, α-smooth muscle actin (α-SMA) and PECAM expression levels were elevated in the corpus cavernosum, indicating that smooth muscle and angiogenesis increased in injured tissues. Figure 3 shows that in the DM ED + SDF-1 eMSC group, SDF-1-Expressing Engineered MSCs had a more positive influence on tissue repair than BM-MSCs.

### 2.4. Increased Expression of neuronal nitric oxide synthase (nNOS) and p-eNOS/eNOS in the Dorsal Penile Nerve

**Figure 4:**
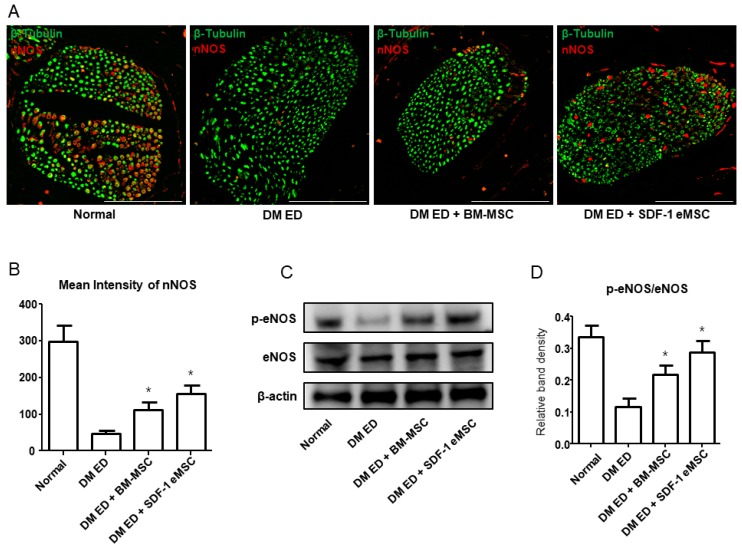
Representative images of neuronal nitric oxide synthase (nNOS) in the dorsal penile nerve. (**A**) Red indicates nNOS and green indicates the dorsal penile nerve. Scale bar: 100 μm. Original magnification: ×400; (**B**) Mean intensity of nNOS for each group; (**C**) Western blot of phosphorylated endothelial nitric oxide synthase (p-eNOS) and eNOS expression in the corpus cavernosum; (**D**) Quantitative results of p-eNOS/eNOS in each group. Each bar shows the mean value (standard deviation). * *p* < 0.05 compared with the diabetes mellitus erectile dysfunction (DM ED) group.

Figure 4A shows that the intensity of neuronal nitric oxide synthase (nNOS) varied among groups. nNOS was lower in the DM ED group than in the other groups (Figure 4B). In a quantitative analysis, intensities were higher in the SDF-1 eMSC group than in the normal (*p* < 0.05) and BM-MSC groups. Figure 4C summarizes p-eNOS and eNOS expression in each group. Figure 4D shows that in the SDF-1 eMSC group, p-eNOS levels were higher than those in the other groups.

### 2.5. High Stromal Cell-derived Factor-1 Expression Increases Fibroblast Growth Factor and Vascular Endothelial Growth Factor Levels In Vivo

**Figure 5:**
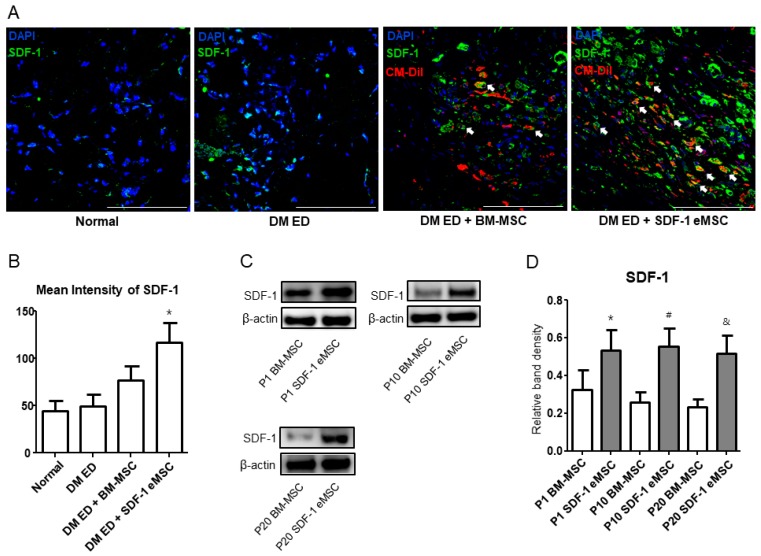
Representative images of SDF-1 expression in vivo and in vitro. (**A**) Representative images of SDF-1 expression in the corpus cavernosum. CM-DiI-labeled MSCs are stained in red and green represents SDF-1. Cell nuclei are stained blue with 4,6-diamidino-2-phenylindole. Arrow indicates SDF-1 released by MSCs. Scale bar: 100 μm. Original magnification: ×200; (**B**) Mean intensity of SDF-1 expression; (**C**) SDF-1 expression after each passage of BM-MSCs in vitro; (**D**) Relative density of bands in the western blot analysis for each group. Each bar shows the mean value (standard deviation). * *p* < 0.05 compared with passage 1 BM-MSCs, ^#^ *p* < 0.05 compared with passage 10 BM-MSCs, and ^&^ *p* < 0.05 compared with passage 20 BM-MSCs.

**Figure 6:**
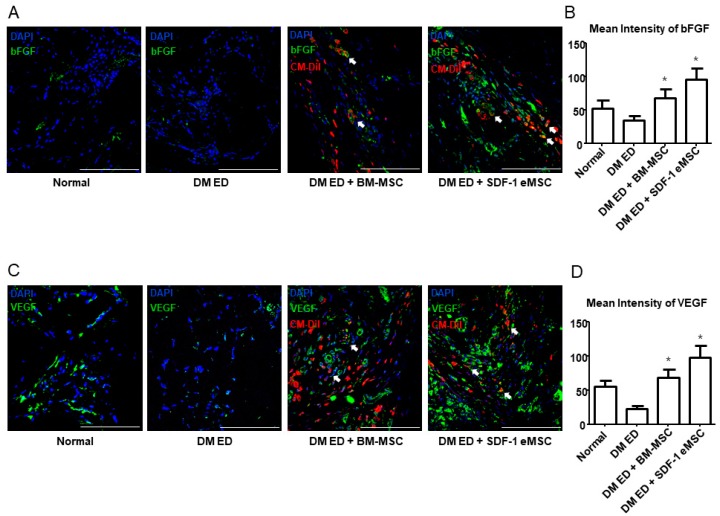
Representative images of bFGF and VEGF expression in the corpus cavernosum. (**A**) Representative images of bFGF staining for each group. Red indicates MSCs, green indicates bFGF, and blue shows cell nuclei. Arrow indicates bFGF released by MSCs. Scale bar: 100 μm. Original magnification: ×200; (**B**) Mean intensity of bFGF expression. (**C**) Representative images of VEGF staining for each group. Red indicates MSCs, green indicates VEGF, and blue indicates cell nuclei. Arrow indicates VEGF released by MSCs. Scale bar: 100 μm. Original magnification: ×200; (**D**) Mean intensity of VEGF expression. Each bar shows the mean value (standard deviation). * *p* < 0.05 compared with the DM ED group.

Figure 5A shows that in the DM ED + SDF-1 eMSC group, SDF-1 expression levels were higher than those of the other groups. Figure 5B summarizes the quantitative analysis (*p* < 0.05). To verify the SDF-1 expression results in each group in vitro, a western blotting analysis of engineered cells and normal cells was performed. As shown in Figure 5C,D, after 10 and 20 passages, the expression of SDF-1 was still higher in the SDF-1 eMSCs group than in the BM-MSC group in vitro (*p* < 0.05). Figure 6 shows that in the two MSC groups, both bFGF and VEGF expression levels were increased. However, in the SDF-1 eMSC group, the expression levels of bFGF and VEGF were higher than those in the BM-MSC group. Though MSC treatment could improve ED, SDF-1 eMSCs were more effective.

### 2.6. MSCs Could Activate the PI3K/AKT Signaling Pathway in the Corpus Cavernosum

**Figure 7:**
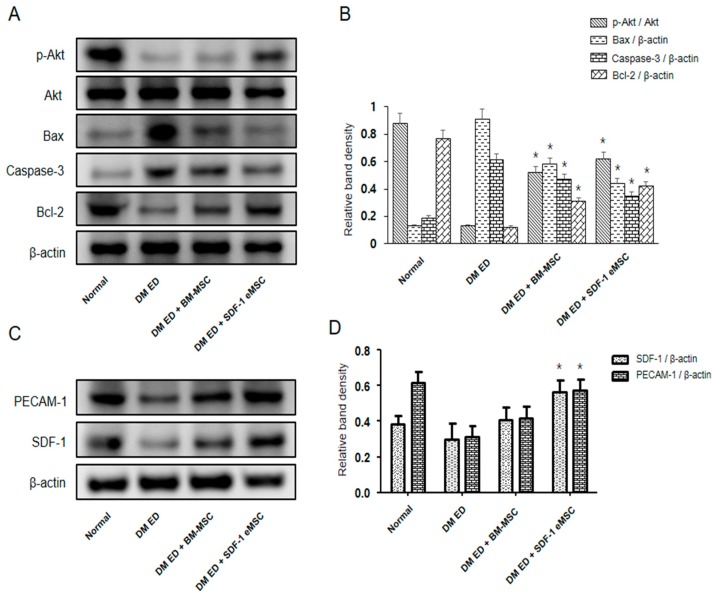
Protein expression in the corpus cavernosum. (**A**) All groups were compared with respect to p-AKT, AKT, Bax, Caspase-3, and Bcl-2 expression in the corpus cavernosum by western blotting. (**B**) Relative density of bands in the western blot analysis for each group. (**C**) All groups were compared with respect to PECAM-1 and SDF-1 levels in the corpus cavernosum by western blotting. (**D**) Relative density of bands in the western blot analysis for each group. Each bar shows the mean value (standard deviation). * *p* < 0.05 compared with the DM ED group.

As shown in Figure 7, in the MSC-treated group, p-AKT/AKT levels were higher (*p* < 0.05), indicating greater PI3K/AKT pathway activation compared with that in the DM ED group. Bcl-2 and Bax are downstream effector proteins of the PI3k/AKT pathway. Bcl-2 levels were higher and Bax levels were lower in the MSC-treated group than in the DM ED group (*p* < 0.05). We also observed lower caspase-3 expression in the DM ED + SDF-1 eMSC group, further supporting the reduction in apoptosis in the corpus cavernosum.

### 2.7. SDF-1-Expressing Engineered MSC Migration and Angiogenic Activity

**Figure 8:**
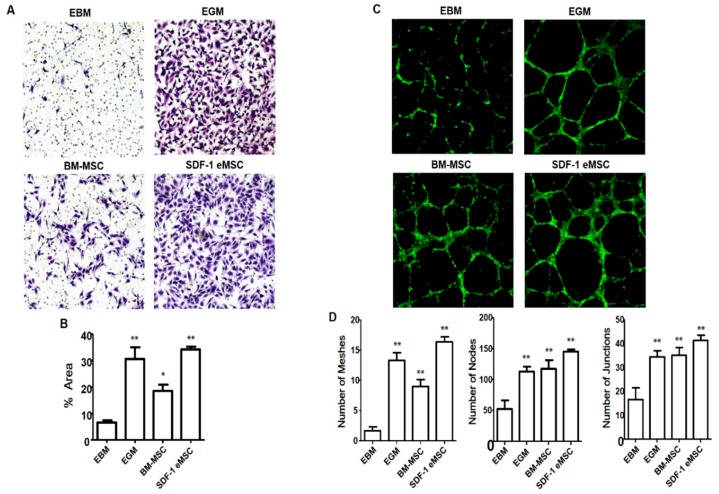
(**A**) Microscopic fields showing migrated HUVECs at the bottom of the PET membrane; (**B**) Bar graph representing the percentage of migrated HUVECs per migrated field in response to BM-MSCs and SDF-1 eMSCs. SDF-1 eMSCs had a greater chemotactic effect than BM-MSCs. Original magnification: ×100; (**C**) Fluorescence microscopy images showing the formation of the tube network on the Matrigel matrix. BM-MSC- and SDF-1 eMSC-treated groups had greater capillary-like structures than EBM-treated groups; (**D**) Quantitative results for total meshes, total nodes, and total junctions in each group. Original magnification: ×50. * *p* < 0.05 compared with the DM ED group. ** *p* < 0.01 compared with the DM ED group.

Endothelial cell migration assay (Figure 8A) revealed greater SDF-1 eMSC migration than that of BM-MSCs. For comparison, the assay was repeated using human umbilical vein endothelial cells (HUVECs). As shown in Figure 8A, SDF-1 eMSCs had a similar tendency to HUVECs with respect to cell migration. These results indicated that under high SDF-1 conditions, BM-MSCs generated more mesh than other cells, similar to the results for HUVECs (Figure 8C).

### 2.8. Expression Levels of SDF-1, VEGF, and bFGF Were Compared between BM-MSCs and Empty Vector Engineered BM-MSCs

**Figure 9:**
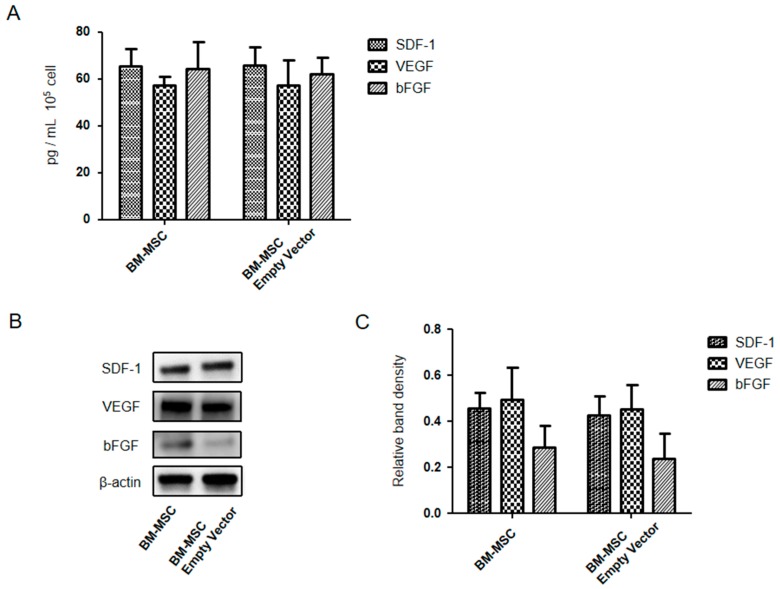
In vitro expression levels of SDF-1, VEGF, and bFGF were compared between BM-MSCs and empty vector engineered BM-MSCs passage 10 by quantitative ELISA and western blotting. (**A**) SDF-1, VEGF, and bFGF concentration in vitro by ELISA; (**B**) Expression levels of SDF-1, VEGF, and bFGF in vitro by western blotting; (**C**) Relative density of bands in the western blot analysis for each group.

Figure 9 shows that the SDF-1, VEGF, and bFGF were compared between BM-MSCs and empty vector engineered BM-MSCs passage 10. In vitro found no significant (*p* < 0.05; *N* = 6) difference as measured by enzyme linked immunosorbent assay (ELISA) and western blotting.

## 3. Discussion

DM ED is generally treated by drug therapy, but a considerable portion of patients are not satisfied with this approach [28]. Accordingly, an alternative approach is needed. ED caused by DM is related to blood vessel spasms and vascular injury due to chronic hyperglycemia [25,26,28,29] in the corpus cavernosum. Therefore, treatment of vascular injury is the key for improving DM ED. Certainly, ED is not a life-threatening disease; therefore, it is difficult to apply genetically modified stem cells in ED patients. Nevertheless, urologists are still striving to find an effective method to address this issue. Recently, Yiou et al. [20] reported a clinical trial that investigated extracavernous injection of MSCs into patients with ED. Stem cells were aspirated from the posterior iliac crest under general anesthesia and injected into both cavernous bodies on the same day. After a 1-year follow-up, they concluded that MSC injection may improve erectile function, which boosted our confidence. A recent study has shown that MSC administration increases VEGF in targeted tissues [27]; the increase in VEGF was attributed to the release of MSCs. However, we wondered whether other factors led to high VEGF expression and why MSCs in injured tissues produced more VEGF. In this study, we found that compared with the BM-MSC group, rats with SDF-1-expressing BM-MSCs had increased vascular smooth muscle contents in the corpus cavernosum. Similar results were obtained in an ICP analysis, indicating that SDF-1 was an important factor in injured tissue recovery and ED improvement.

Liang et al. [30] suggested that the main limitation of MSC treatment was the inability of transplanted MSCs to accurately accumulate at the therapeutic target and function effectively. A previous study [22] has shown that injected MSCs migrate to the circulatory system and disperse via the blood flow; only a few MSCs gather at injured tissues, below the targeted therapeutic concentrations. By labelling MSCs with a fluorescent dye, Cell Tracker™ CM-DiI [31], we found that after injection, a large number of MSCs gathered in the corpus cavernosum in the treatment group, proving that MSCs migrated to the target tissues. Studies have shown that SDF-1 levels are high during cell proliferation and tissue repair [30,32], and these chemotactic factors could promote the accumulation of these functional cells at injured tissues [33]. In our study, we showed that BM-MSCs expressing high levels of SDF-1 could release more SDF-1 than normal BM-MSCs. We found increased VEGF expression and angiogenesis in the upgraded SDF-1-expressing BM-MSC group compared with the other groups, explaining why ED rats recovered more effectively after SDF-1-expressing BM-MSC treatment. Fandel et al. [22] showed that in cavernous nerve-injured ED rats, SDF-1 could drive injected stem cells to the major pelvic ganglia for neural restoration. Yamaguchi et al. [23] found that SDF-1 could drive endothelial progenitor cells to injured tissues and improve angiogenesis.

Chemotaxis of SDF-1 could drive both exogenous and endogenous stem cell recruitment. Thus, VEGF expression in injured tissues should be greater than that in MSCs, and other endogenous stem cells could gather at the targeted tissues for vascular repair. We found differential expression of SDF-1 among the groups. In the SDF-1-expressing BM-MSC group, VEGF expression was higher than that in the normal MSC group. Since the MSCs were equal and VEGF differed between the two groups, we inferred that SDF-1 played a key role in the process of ED improvement. These results indicated that SDF-1 expressed by the injected BM-MSCs drove endogenous cells to targeted tissues. These endogenous cells could accelerate angiogenesis and tissue repair by releasing VEGF, as confirmed by our results.

We performed preliminary studies to verify whether or not empty vector engineered MSCs differed from un-engineered MSCs regarding their ability to affect and influence the outcome of our in vivo results. We tested these cells for the production of SDF-1, VEGF, and bFGF in vitro and found no significant difference as measured by ELISA and western blotting. In addition, we performed western blot analysis for the same factors and found no difference with the exception that bFGF was slightly lower in the empty vector engineered BM-MSCs. From these findings, we determined that un-engineered MSCs could serve as a good control, and thus, it was not necessary to continue studying empty vector engineered MSCs as a control in our in vivo studies. Furthermore, previous studies showed that empty vector engineered adipose stem cells had no notable effect on chondrogenesis [34].

De Bock et al. [35] suggested that glycolysis provided energy to endothelial cells for proliferation, even in conditions of sufficient oxygen. In new tissues, vascular growth was promoted by continuous budding. At the end of the budding, the blood supply was insufficient to support angiogenesis, and endothelial cells obtained ATP via anaerobic glycolysis. Similarly, in the corpus cavernosum of DM ED, vascular injury and vasospasm caused by hyperglycemia lead to an insufficient blood supply. However, endothelial cells still obtain enough ATP to support proliferation for vascular repair via anaerobic glycolysis. Han et al. [36] believed that p-AKT could stimulate endothelial cells to produce substantial eNOS, which was the key factor for vasodilatation. In this study, we found higher levels of eNOS in the corpus cavernosum of the treatment group than in the untreated group, and p-AKT/AKT showed a similar trend. It is possible that MSCs could produce high levels of VEGF in the corpus cavernosum, which combined with the surface receptors of endothelial cells to trigger the PI3K-AKT signaling pathway, thereby increasing eNOS. A large quantity of eNOS would stimulate vasodilatation and inhibit vasospasm, which could recover the blood supply and improve ED.

Stem cell therapy is beneficial for ED caused by nerve injury [37]. Stem cells could promote nerve recovery. nNOS produced by nerve cells is not only involved in the penis erection reflex but also plays key roles in angiogenesis and vasodilatation [38]. Wang et al. [39] found that MSCs could stimulate nerve cells to produce nNOS, which improved angiogenesis in the injured tissues. In this study, we obtained similar results; in the corpus cavernosum of the treatment group, nNOS levels were higher than those in the untreated group, indicating that MSCs could promote nerve cell production of nNOS in vivo for angiogenesis. After MSC transplantation, hypoxemia and the oxidative stress response caused by hyperglycemia decreased the survival rate of injected MSCs, and the short lifespan of MSCs after intravenous infusion raised doubt about the contribution of growth factors secreted by MSCs to the modulation of vascular repair and cellular proliferation [40,41]. Bcl-2 could inhibit apoptosis, and downgraded Bax could decrease the rate of apoptosis [42]. In our study, we found activation of the PI3K-AKT pathway in the corpus cavernosum, which was thought to stimulate the expression of Bcl-2 and inhibit the expression of Bax. We found significant Bcl-2 and Bax expression in the corpus cavernosum in all groups. We inferred that the activation of the PI3K-AKT pathway by VEGF increased the expression of Bcl-2 and decreased that of Bax, which improved the survival rate of the transplanted MSCs.

We further tested upstream factors and downstream effector proteins, which did not block the signaling pathway, to verify our findings.

## 4. Materials and Methods

### 4.1. SDF-1-Expressing MSCs and Experimental Animal Preparation

Primary bone marrow mesenchymal stem cells (BM-MSCs) were cultured in low glucose-containing Dulbecco’s modified Eagle’s medium (Gibco, Gaithersburg, MD, USA) supplemented with 20% fetal bovine serum (FBS; Gibco) and 5 ng/mL basic fibroblast growth factor (bFGF; Cell Signaling Technology, Danvers, MA, USA) at 37 °C and 5% CO_2_, and engineered BM-MSCs were cultured with 10% FBS.

To generate engineered BM-MSCs, *c-myc*, *hTERT*, tetracycline transactivator (*tTA*), and *SDF-1* genes were synthesized and transfected using the pBD lentiviral vector (SL BIGEN, Seongnam, Korea). Transfected engineered BM-MSCs were selected by antibiotics. Selected engineered BM-MSCs were isolated to obtain a monoclonal cell population by the limiting dilution method. Final monoclonal cells were selected based on SDF-1 expression, proliferation, and other MSC phenotypes. Before in vivo injection, SDF-1-expressing engineered BM-MSCs (SDF-1 eMSCs) were irradiated.

The reference sequence for SDF-1 was NM_000609.6. We introduced an optimized DNA sequence into the vector (GenScript, Nanjing, China). DNA sequencing was performed to confirm that the reading frame of the introduced sequence was correct (Cosmo Genetech, Seoul, Korea). Cryopreserved cells were thawed and seeded onto 12-well plates at a density of 1 × 10^5^ cells per well. After 48 h, the supernatant was harvested and SDF-1 protein levels were measured by ELISA (R&D Systems, Minneapolis, MN, USA); the expression level of SDF-1 was about 10 ng/mL.

Fifty 8-week-old male Sprague–Dawley rats weighing about 250–300 g were purchased from Orient Bio Co. (Seongnam, Korea). All animal experiments in this study were approved by the Institutional Animal Care and Use Committee of the Catholic University of Korea (CUMC-2016-0218-01, 31 August 2016). All surgeries were performed under chloral hydrate anesthesia, and all efforts were made to minimize animal suffering.

### 4.2. Establishment of DM ED Models and BM-MSC Administration

After fasting, 12 rats were randomly chosen for the normal group (control) and intraperitoneally injected with saline. In the other rats, ED was induced by an intraperitoneal injection of streptozotocin (STZ; Sigma Aldrich, St. Louis, MO, USA) at a dose of 60 mg/kg body weight. At 72 h after STZ injection, DM was confirmed by blood glucose levels; blood samples were obtained from tail veins, and only rats with a fasting blood glucose level of ≥300 mg/dL were selected as diabetic and used for further tests (*n* = 36). The rats were randomly divided into 4 groups: a normal group (control, *n* = 12), a DM ED group (*n* = 12), a DM ED + BM-MSC group (*n* = 12), and a DM ED + SDF-1 eMSC group (*n* = 12).

### 4.3. BM-MSC Treatment

Four weeks after DM ED was induced, rats in the DM ED + BM-MSC group or DM ED + SDF-1 eMSC group were treated with BM-MSCs or SDF-1 eMSCs by bilateral intracavernous injection under anesthesia (1 × 10^6^ BM-MSCs diluted in phosphate-buffered saline). Rats in the control group were injected with an equal volume of saline. To track the location of BM-MSCs, they were labeled with a fluorescent dye (Cell Tracker™ CM-DiI; Molecular Probes, Eugene, OR, USA) according to the manufacturer’s protocol.

### 4.4. Measurement of Erectile Function

After treatment for 4 weeks, all rats were tested for erectile function by measuring intracavernous pressure (ICP) and mean arterial pressure (MAP) under anesthesia. A 23-gauge butterfly needle containing 250 U/mL heparin solution was carefully inserted into the proximal corpus cavernosum and the other end of the PE-50 tube was connected to a pressure transducer (Grass model S48 K; Astro-Med Inc., West Warwick, RI, USA) to measure the ICP. A bipolar stainless steel electrical stimulator was used to stimulate the major pelvic ganglion at 10 V for 50 s and 2.4 mA with a pulse width of 2.5 ms. The maximum ICP value of three stimulations was used for the statistical analysis in each rat. ICP was normalized to MAP, which was recorded using a BD Intramedic PE-50 tubing (BD, Franklin Lakes, NJ, USA) inserted into the left carotid artery at the same time. After the measurement of erectile function, rats were sacrificed and the penises were harvested for immunohistochemistry and western blot analyses.

### 4.5. Histochemistry

The collected cavernous nerve and penis samples were fixed in 4% paraformaldehyde for 24 h at 4 °C before creating a paraffin block. The following primary antibodies were used: Neuronal nitric oxide synthase (nNOS, diluted 1:200; Santa Cruz Biotechnologies, Santa Cruz, CA, USA), alpha smooth muscle actin (α-SMA, diluted 1:500; Abcam, Cambridge, UK), vascular endothelial growth factor (VEGF; diluted 1:200; Santa Cruz Biotechnologies), basic fibroblast growth factor (bFGF, diluted 1:500; Cell Signaling Technology), stromal cell-derived factor-1 (SDF-1 diluted 1:200; Abcam), and platelet endothelial cell adhesion molecule (PECAM-1, diluted 1:500; Abcam), and 6-diamidino-2-phenylindole (DAPI; Vector Laboratories, Inc., Burlingame, CA, USA) was used to stain nuclei. Digital images were obtained using a Zeiss LSM 510 Meta confocal microscope (Zeiss, Oberkochen, Germany), and the mean intensity was calculated using ZEN 2012 (Zeiss).

### 4.6. Western Blotting

The collected corpus cavernosum tissue was homogenized using ice-cold RIPA buffer (Cell Signaling Technology) containing ethylene diamine tetra acetic acid-free protease inhibitor cocktail and phosphatase inhibitor cocktail (Roche Diagnostics GmbH, Basel, Switzerland), and particulate mass was removed by centrifugation (15,000× *g*) for 15 min at 4 °C. Supernatants were analyzed by SDS-PAGE. Primary antibodies against endothelial nitric oxide synthase (eNOS diluted 1:200; Cell Signaling Technology), phosphorylated endothelial nitric oxide synthase (p-eNOS diluted 1:200; Cell Signaling Technology), AKT (diluted 1:200; Cell Signaling Technology), p-AKT (diluted 1:200; Cell Signaling Technology), Bcl-2 (diluted 1:200; Cell Signaling Technology), Bax (diluted 1:200; Cell Signaling Technology), SDF-1 (diluted 1:500; Abcam), PECAM (diluted 1:200; Abcam), Caspase-3 (diluted 1:400; Cell Signaling Technology), VEGF (diluted 1:500; Abcam), bFGF (diluted 1:500; Abcam), and β-actin (diluted 1:1000; Abcam) were used.

### 4.7. BM-MSC and SDF-1 eMSC Preparation in Conditioned Media

BM-MSCs and SDF-1 eMSCs were seeded in a 100 mm culture dish. After fully attaching, completed growth media were removed and cells were washed with PBS 3 times. Subsequently, 10 mL of fresh serum-free basal medium was added, followed by incubation at 37 °C in 5% CO_2_ for 3 days. Collected media were centrifuged at 3000 rpm at 4 °C for 15 min. The supernatant was collected and SDF-1 eMSC conditioned media were stored at −80 °C until further analyses.

### 4.8. Tube Formation Assay

A total of 200 μL of BD Growth Factor Reduced (GFR) Matrigel Basement Membrane Matrix (BD Biosciences) was added to a 24-well plate and incubated at 37 °C for 30 min. HUVECs (1 × 10^5^ cells/well) were incubated on a plate coated with Matrigel in endothelial growth media (EGM), endothelial basal media (EBM) (no growth supplement), and EBM with 30% conditioned media (BM-MSC and SDF-1 eMSC). After 12 h, cells were stained using calcein AM dye (2 µg/mL). Tube formation was observed using a fluorescence microscope (Axio200; Carl Zeiss). Images were traced and skeletonized using an image and angiogenesis tool. The total number of meshes, nodes, and junctions were quantified for each skeleton.

### 4.9. Endothelial Cell Migration Assay

The spontaneous formation of capillary-like structures by HUVECs on the Matrigel basement membrane matrix was evaluated to assess angiogenesis. A total of 5 × 10^4^ HUVECs in 400 µL of EBM (no growth supplement) were plated in the upper compartment, and 600 µL of medium was added to the lower chamber (EGM, EBM, EBM with BM-MSC, and SDF-1 eMSC conditioned media). The plates were then cultured for 8 h under normal conditions. To estimate the number of migrated cells, the inserts were fixed with 4% PFA and the upper surface of the Transwell membranes was gently swabbed with cotton to remove non-migrated cells and stained with crystal violet (0.1%). The cells that traversed the membrane were counted by bright-field microscopy.

### 4.10. ELISA

The concentration of SDF-1, VEGF and bFGF were measured by species-specific immunoassay ELISA kits (R&D Systems Europe, Abingdon, UK) according to the manufacturer’s instructions. BM-MSCs and empty vector engineered BM-MSCs at passage 10 were cultured for 12 h in a constant-temperature incubator, and then 1 mL of the medium was collected. Absorbance was read at a wavelength of 450 nm using a microplate reader (Synergy H1 M, Biotek, Winooski, VT, USA).

### 4.11. Statistical Analysis

All data are presented as means ± standard error (SD) and were analyzed using SPSS version 22.0 (IBM, Armonk, NY, USA). Student’s *t*-tests, one-way ANOVA, and 2 × 2 factorial ANOVA were used, as appropriate, to evaluate differences among groups. *p* < 0.05 was considered statistically significant.

## 5. Conclusion

Our results proved that SDF-1 eMSCs released chemotactic factors, which could drive MSCs to migrate to targeted tissues, thereby increasing angiogenesis and effectively improving ED. Additionally, high SDF-1 expression substantially decreased apoptosis in the corpus cavernosum.

## Figures and Tables

**Table 1:** Body weights and serum glucose levels.

	Pre-DM	After 4 Weeks
**Body Weight (g)**		
Normal (*n* = 12)	251.6 ± 8.5	311.3 ± 13.6
DM ED (*n* = 12)	254.1 ± 9.7	159.6 ± 16.7 *
DM ED + BM MSC (*n* = 12)	259.2 ± 10.9	170.7 ± 12.8 *
DM ED + SDF-1 eMSC (*n* = 12)	249.8 ± 10.3	183.6 ± 7.2 *^,#^
	**Pre-DM**	**After 4 Weeks**
**Serum Glucose (mg/dL)**		
Normal (*n* = 12)	123.6 ± 3.3	121.7 ± 1.9
DM ED (*n* = 12)	123.8 ± 2.9	392.2 ± 8.7 *
DM ED + BM MSC (*n* = 12)	124.1 ± 3.8	383.9 ± 9.6 *
DM ED + SDF-1 eMSC (*n* = 12)	122.5 ± 3.2	376.8 ± 5.9 *^,#^

* Significant difference (*p* < 0.05) compared with the Normal group. ^#^ Significant difference (*p* < 0.05) compared with the DM group. The serum glucose levels were measured at fasting status. SDF-1: stromal cell-derived factor-1; DM ED: diabetes mellitus erectile dysfunction.

## Data Availability

The data used to support the conclusions of this study are available from the corresponding author upon request.
